# Retraction Note: Berberine reduces temozolomide resistance by inducing autophagy via the ERK1/2 signaling pathway in glioblastoma

**DOI:** 10.1186/s12935-024-03394-2

**Published:** 2024-06-07

**Authors:** Huiling Qu, Xiaofu Song, Zhuyin Song, Xin Jiang, Xin Gao, Lijuan Bai, Jiao Wu, Li Na, Zhicheng Yao

**Affiliations:** 1 Department of Neurology, The People’s Hospital of Liaoning Province, 33 Wenyi Road, Shenhe District, Shenyang, Liaoning 110016 China; 2 Department of Laboratory Medicine, The People’s Hospital of Liaoning Province, Shenyang, Liaoning China

**Retraction Note: Cancer Cell International (2020) 20:592**

10.1186/s12935-020-01693-y.

The Editor in Chief has retracted this article. Following publication concerns were raised regarding a number of figures, including but not limited to:

- The TMZ 0hr panel in Fig. 2a appears similar to the FDPs 0h panel in Fig. 3 in [1] and the BRD4-KO#1 0h panel in Fig. 2C in [2].
- The Vector and CtBP1 bands in Fig. 3B appear similar to U251 Vector and b-catenin bands in Fig. 6G in [3] and p62 and b-actin blots in Fig. 3B in [4].
- The p-62 u87/TMZ-R in Fig. 3E appears similar to the Mcl-1 CHX blot in Fig. 4C in [5] and the p-PI3K GIST430 in [2].

The Editor-in-Chief therefore no longer has confidence in the results and conclusions of this article. The authors did not state explicitly whether they agree to this retraction.
